# Trajectories of Cognitive Complaints in Patients With Breast Cancer and Their Association With Psychosocial and Neurobiological Factors

**DOI:** 10.1002/cam4.71130

**Published:** 2025-08-07

**Authors:** Rob Colaes, Gwen Schroyen, Rebeca Alejandra Gavrila Laic, Jeroen Blommaert, Sigrid Hatse, Ann Smeets, Stefan Sunaert, Sabine Deprez

**Affiliations:** ^1^ Department of Imaging and Pathology KU Leuven Leuven Belgium; ^2^ Leuven Brain Institute KU Leuven Leuven Belgium; ^3^ Leuven Cancer Institute KU Leuven Leuven Belgium; ^4^ Department of Oncology KU Leuven Leuven Belgium; ^5^ Department of Oncology, Surgical Oncology University Hospitals Leuven Leuven Belgium; ^6^ Department of Radiology University Hospitals Leuven Leuven Belgium

**Keywords:** clustering, cognitive complaints, CRCI, longitudinal trajectories, psychosocial, Rs‐fMRI breast cancer

## Abstract

**Introduction:**

Cancer‐related cognitive impairment (CRCI) is common among patients with breast cancer, with significant variability in both severity and duration of cognitive complaints. This study aimed to elucidate this variability by identifying distinct trajectories of cognitive complaints over time in patients with breast cancer. Additionally, we explored the relationships between these trajectories and various clinical, demographic, psychosocial, neuropsychological, neuroimaging (resting‐state functional MRI, rs‐fMRI), and serum markers.

**Methods:**

In this prospective study, 67 patients with non‐metastatic breast cancer underwent psychosocial questionnaires, neuropsychological testing, rs‐fMRI, and serum markers at diagnosis (T0), 8 months after diagnosis (T1), and 16 months after diagnosis (T2). Using the partition around medoids (PAM) algorithm on the difference scores of cognitive complaints, patients were clustered into distinct groups. Differences between the groups were assessed using linear mixed effects models. Rs‐fMRI was analyzed using whole‐brain graph theory and connectivity in four cognition‐related networks.

**Results:**

Four different trajectories of cognitive complaints were identified: stable with no changes in complaints (*n* = 24), improving with a decrease in complaints at T1 (*n* = 15), a short‐term affected with an increase in complaints at T1 and recovery at T2 (*n* = 13), and a long‐term affected with an increase in complaints at T1 and T2 (*n* = 15). While the groups strongly correlated with changes in the psychosocial measures, only subtle associations were found with neuropsychological tests, serum markers, or rs‐fMRI analyses.

**Conclusions:**

This research affirmed the presence of distinct trajectories of cognitive complaints in patients with breast cancer, which were associated with differences in anxiety, fatigue, stress, and depression.

## 1 Introduction

Breast cancer is the most common cancer in women globally, but survival rates have strongly increased due to diagnostic and therapeutic improvements [1, 2]. Nevertheless, up to 75% of cancer patients exhibit cancer‐related cognitive impairment (CRCI) during and after treatment [3, 4]. Objective cognition, measured by neuropsychological tests, has revealed cognitive deficiencies in the domains of memory, attention, processing, and executive functioning in patients with cancer [5, 6, 7, 8]. In addition, self‐reported measures indicate that patients experience cognitive complaints mostly related to memory and executive functioning [9, 10]. This is often associated with psychosocial factors including anxiety, depression, fatigue, stress, and sleep quality [11, 12, 13, 14, 15].

Neuroimaging modalities have revealed both structural and functional brain alterations in patients with breast cancer, often associated with cognitive decline [16, 17, 18]. For instance, functional connectivity within cognition‐related networks has been studied through resting‐state functional MRI (rs‐fMRI) in patients with breast cancer. By analyzing the connectivity between certain regions of interest (ROI‐to‐ROI analysis), hypo‐connectivity was shown within the default mode network, the salience network, the fronto‐parietal network, and the dorsal attention network [19, 20, 21]. Using graph analysis, the topological organization of functional brain networks can be studied [22]. Lower network segregation and integration have been observed in patients with breast cancer [23, 24, 25], which has been associated with memory dysfunction [24].

The neurobiological pathways causing these cognitive dysfunctions and brain alterations remain unclear. Different mechanisms have been suggested, including induced hormonal changes, treatment‐related direct neurotoxicity, DNA damage, genetic predisposition, and indirect immune‐mediated inflammatory processes [26, 27, 28]. Peripheral proinflammatory cytokines accumulating as a result of the cancer and its treatment may cross the blood–brain barrier, potentially causing neuroinflammation and brain damage. Chemotherapy, though generally unable to cross the BBB, might facilitate cytokine passage [29]. Chemotherapy‐induced changes in inflammatory markers have been linked to cognitive decline in cancer survivors, from treatment initiation until years post‐treatment [30].

Importantly, there is considerable heterogeneity in the severity of cognitive dysfunctions, with variations in both the duration and the specific cognitive domains affected [31, 32]. While some patients are unaffected, others may experience cognitive impairment but eventually recover after a while [33] and yet others may even exhibit sustained cognitive deficiencies long after treatment [16, 34]. Unfortunately, the factors underlying this heterogeneity remain largely unclear. A better understanding of the key determinants of worse cognitive outcomes, including the identification of predictive markers, could significantly improve the development of personalized treatments and the overall quality of life for cancer survivors [35, 36].

However, studies often overlook the underlying heterogeneity in CRCI by using a dichotomous, impaired or not, definition or by comparing treatment groups without considering cognitive variability, leading to oversimplification [35]. A potential solution to this issue is to classify patients into newly defined subgroups of CRCI, with different severity and timing of cognitive decline. Data‐driven approaches, such as latent class models and unsupervised clustering, have been used in epidemiological studies, as well as in CRCI studies [35, 37]. For instance, a prospective study identified three subgroups of cognitive complaints in patients with breast cancer using a latent class growth analysis. Worse cognitive classes were associated with psychosocial variables, including depression, anxiety, and sleep deprivation [38]. Another approach could be the incorporation of neurobiological data into defining cognitive functioning [39]. In fact, a recent study has used a combination of rs‐fMRI connectivity patterns and neuropsychological tests to define CRCI subtypes in patients with breast cancer. The resulting subtypes significantly differed in cognitive performance, regional connectivity, psychological distress, sleep deprivation, and cognitive resilience [40, 41]. These studies confirm the heterogeneity in cognitive decline, highlighting the need for a broader definition of CRCI. However, most research only compares clinical, demographic, and psychosocial factors among subtypes, overlooking key determinants of CRCI heterogeneity. Characterizing distinct brain connectivity patterns and inflammatory profiles among CRCI subtypes may identify novel risk markers and guide personalized interventions.

In this longitudinal study, we examined (1) the presence of distinct trajectories of cognitive complaints over time in patients with breast cancer, by employing the data‐driven partitioning around medoids (PAM) algorithm, and (2) we explored how these trajectories relate to various clinical, demographic, psychosocial, neuropsychological, rs‐fMRI, and inflammatory serum markers.

## 2 Methods

### 2.1 Participants

**FIGURE 1:**
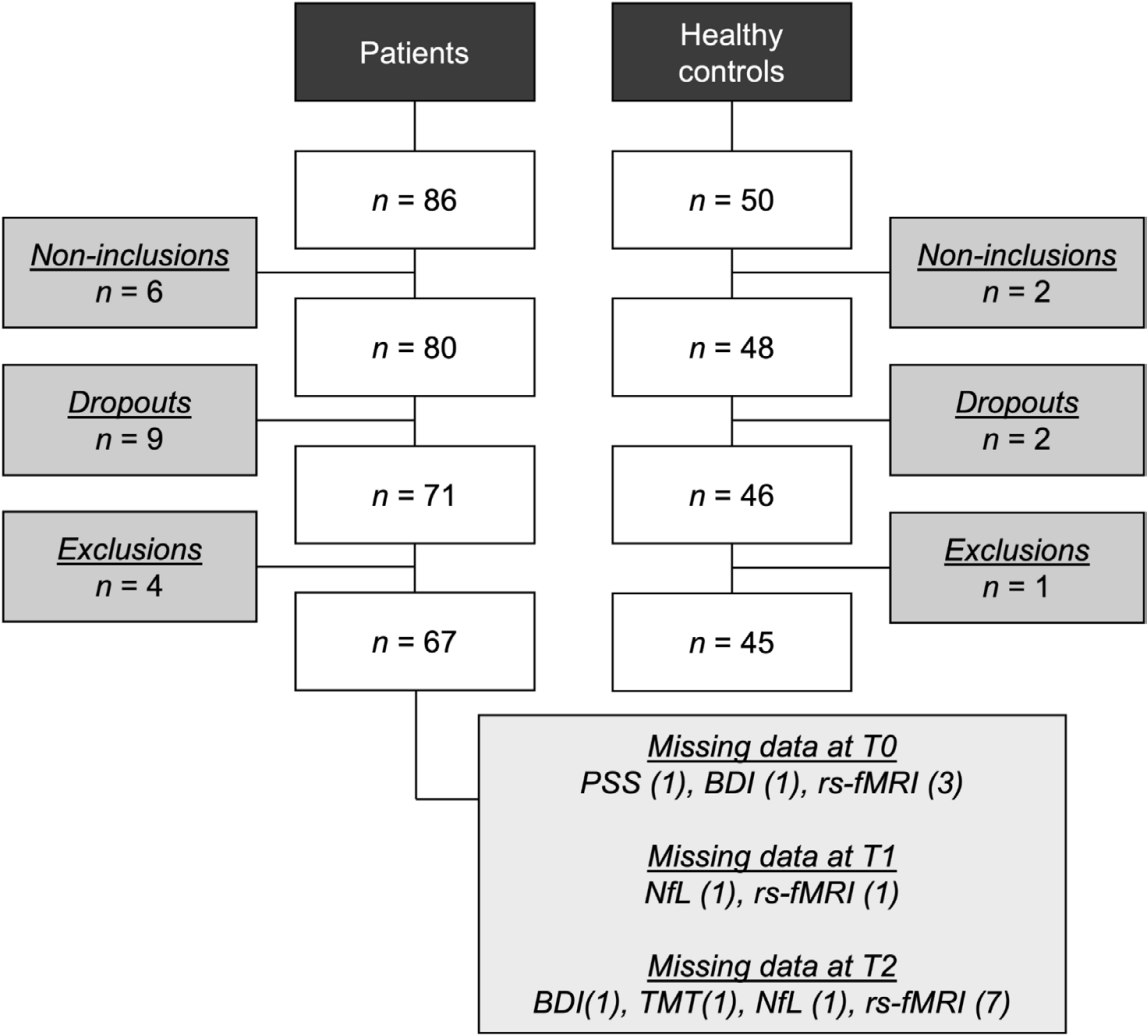
Flowchart of study participant inclusion. Non‐inclusions refer to recruited participants that did not start the study. Dropouts and non‐inclusions occurred due to withdrawal, Covid‐19 restrictions, discomfort in the scanner, and age‐matching of patients with healthy controls. The exclusions within this study were due to changes in treatment and/or discovery of neurological conditions during scanning (very high number of white matter lesions). Some patients had missing modalities at one or two time‐points due to time constraints, fear of the MRI, or forgetting to complete a questionnaire. BDI, Beck's depression inventory; NfL, neurofilament light chain; PSS, perceived stress scale; rs‐fMRI, resting‐state functional MRI; TMT, trail making test.

This prospective study recruited women aged ≤ 65 years diagnosed with primary non‐metastatic breast cancer from December 2018 to June 2021 through the Multidisciplinary Breast Center (MBC) at University Hospitals Leuven (Belgium). For a subset of this cohort, an analysis on neurofilament light‐chain has been reported earlier [42]. The study included 67 women diagnosed with non‐metastatic breast cancer who were treated without chemotherapy or who received (neo)adjuvant chemotherapy, consisting of four rounds of epirubicin at 90 mg/m^2^ and cyclophosphamide at 600 mg/m^2^, as well as four to twelve rounds of paclitaxel at 80 mg/m^2^. Ten patients received additional rounds of chemotherapy. All patients underwent surgery, and some received additional treatment in the form of radiotherapy, targeted therapy, and/or endocrine therapy (Table 2). Additionally, healthy controls (HC) were recruited through online advertisements and matched at the group level to patients on gender, age, and education. To evaluate short‐ and long‐term neurocognitive changes, participants were assessed at three time points: at diagnosis (T0), 8 months after diagnosis (T1), and 17 months after diagnosis (T2). Healthy controls (HCs) were assessed at matched time points. For patients receiving chemotherapy, the T0 assessment took place after surgery or when scheduled for neoadjuvant chemotherapy, before the start of chemotherapy. The T1 assessment took place 3 months post‐chemotherapy. For patients not receiving chemotherapy, the T0 assessment was conducted after surgery. At each time point, neuropsychological tests, questionnaires, blood sampling, and MRI scans were completed on the same day. Only participants who completed the assessment at all three time points were included in the analysis (Figure 1). Women were excluded from the study if they had MRI contraindications, a history of cancer treatment, drug or alcohol abuse, psychiatric/neurological condition/injury, intellectual disability, or systemic steroid use.

### 2.2 Neuropsychological Tests and Self‐Reported Questionnaires

Self‐reported questionnaires on cognitive complaints and psychosocial measures were completed by participants at each time point, alongside a one‐hour neuropsychological test battery, following the guidelines of the International Cognition and Cancer Task Force (ICCTF) [44].

Cognitive complaints were assessed by the Cognitive Failure Questionnaire (CFQ), which consists of 25 questions on cognitive problems in daily activities [45]. Higher scores indicate more cognitive problems, with scores ranging from 0 to 100 (*μ*_norm_ = 31.8, *σ*_norm_ = 11.1) [46]. The total CFQ score was used as the outcome measure for the clustering algorithm.

The psychosocial measures included the Beck Depression Inventory (BDI) [47], the Fatigue Assessment Scale (FAS) [48], the State–Trait Anxiety Inventory (STAI) [49], and the Perceived Stress Scale (PSS) [50].

Based on the recommendations of the ICCTF and neuropsychological tests showing significant differences in our previous work [51], a subset of seven outcomes were used. For the domain of information and visuomotor processing speed: Grooved Pegboard non‐dominant hand (ntDom) [52], Wechsler Adult Intelligence Scale III numbers and letters maximum (WAISCL) [53], and Trail Making Test (TMT) [54]. For memory: Wechsler Adult Intelligence Scale III digit span backwards (WAISd) [53], Auditory Verbal Learning Test sum (AVLTsum), and Auditory Verbal Learning Test delayed recall (AVLTd) [55]. For executive functioning: Controlled Oral Word Association Test (COWA) [56].

### 2.3 Serum Markers

Serum neuroinflammatory markers visin‐like protein‐1 (VILIP‐1), monocyte chemoattractant protein‐1 (MCP‐1), soluble triggering receptor expressed on myeloid cells (sTREM‐1 and sTREM‐2), brain‐derived neurotrophic factor (BDNF), vascular endothelial growth factor (VEG‐F), interleukins (IL‐6 and IL‐18), tumor growth factor‐β (TGF‐β), tumor necrosis factor‐ɑ (TNF‐ɑ), soluble receptor for advanced glycation‐end products (sRAGE), β‐nerve growth factor (β‐NGF), and fractalkine (CX3CL1) were analyzed using multiplex cytometric bead array assays (LEGENDplex Human Neuroinflammation Panel 1, BioLegend, San Diego) according to the manufacturers' instructions. Additionally, serum neurofilament light chain (NfL) was assessed with an enzyme‐linked immunosorbent kit (UmanDiagnostics, Umea) [57] and quantified with an electrochemiluminescent assay [58]. VILIP‐1, TGF‐β, and β‐NGF were excluded due to high interplate variability. A detailed overview can be found in the Supporting Information.

### 2.4 Magnetic Resonance Imaging

MRI data was collected on the same 3T Philips Achieva scanner with a 32‐channel phased‐array head coil at each time point. We collected a high‐resolution T1‐weighted image (MPRAGE, voxel size = 0.8 × 0.8 × 0.8 mm, TR/TE = 5.8/2.5 ms, FA = 8°, FOV = 320 × 320 × 208) and resting‐state functional images (using T2*‐weighted Echo‐planar imaging, resolution = 2 × 2 × 2 mm, FOV = 208 × 208 × 144 mm, FA = 60°, TE/TR = 32/900 ms, acquisition time = 6 min, 400 vol + 4 initial dummy volumes). An overview of the preprocessing and analyses can be found in the Supporting Information. Two separate analyses were performed on the resting‐state functional images: ROI‐to‐ROI and graph theory analysis. For the ROI‐to‐ROI analysis, the default mode, salience, fronto‐parietal, and dorsal attention networks were used (Table S2). For the graph theory analysis, whole‐brain measures of clustering coefficient, global efficiency, local efficiency, and characteristic path length were extracted.

### 2.5 Clustering Algorithm

Different trajectories of cognitive complaints, measured by CFQ, were identified using the Partition Around Medoids (PAM) algorithm in R version 4.2.3 (clusters package). PAM is a robust unsupervised clustering technique that groups data points into clusters by selecting representative points, called medoids, and minimizing a distance measure between each point and its closest medoid [59, 60]. Unlike K‐means, PAM uses actual data points as representation of a cluster, making it less sensitive to outliers [61]. In this analysis, Euclidean distance was used as a distance measure. The difference scores of CFQ between the time points (T1‐T0, T2‐T1) were used for the clustering algorithm to have a partitioning that focuses on the change of cognitive complaints. The optimal number of clusters was determined by comparing 2–5 clusters on average silhouette width, maximal dissimilarity, average dissimilarity, group sizes of > 10%, and the presence of distinct trajectories.

Healthy controls (*n* = 45) were included in the PAM algorithm to provide additional CFQ data points. They were also included when selecting the optimal number of clusters. However, in the analysis of the cluster profiles and longitudinal differences of the identified clusters, healthy controls were not included.

### 2.6 Statistics

Cluster profiles, defined as the characteristics of the patients within each group, were compared using analysis of variance (ANOVA) for continuous variables and Fisher's exact test for categorical variables. Longitudinal differences between the clustered groups were examined using linear mixed effects models with T0 and the group with the most stable trajectory as the references. The models included the outcome parameter as a dependent variable, a random subject intercept, time‐point, group, and the interaction between time‐point and group as fixed effects. Age was included as a covariate in all models, while serum markers included both age and days in storage as covariates [62]. The interaction term between time‐point and group was used to examine whether the rate of change over time differed between the groups. To evaluate the rate of change within each group over time, we conducted a post hoc within‐group analysis. To facilitate interpretation and comparability of effect sizes across all linear mixed models, standardized coefficients were reported in addition to the raw estimates [63]. Given the exploratory nature of this study, corrections for multiple comparisons were not applied. Statistical significance was inferred at *p* < 0.05. Statistical analyses were performed in R (v4.2.3).

## 3 Results

### 3.1 Participants

This study included 67 patients with breast cancer. All women were aged between 31 and 64 (*μ* = 50, *σ* = 8). Chemotherapy was administered to 32 patients (48%). Key patients' characteristics can be found in Table 2. Additionally, 45 HCs were included in the clustering algorithm, with their characteristics provided in the Table S1.

### 3.2 Optimal Number of Trajectories

**FIGURE 2:**
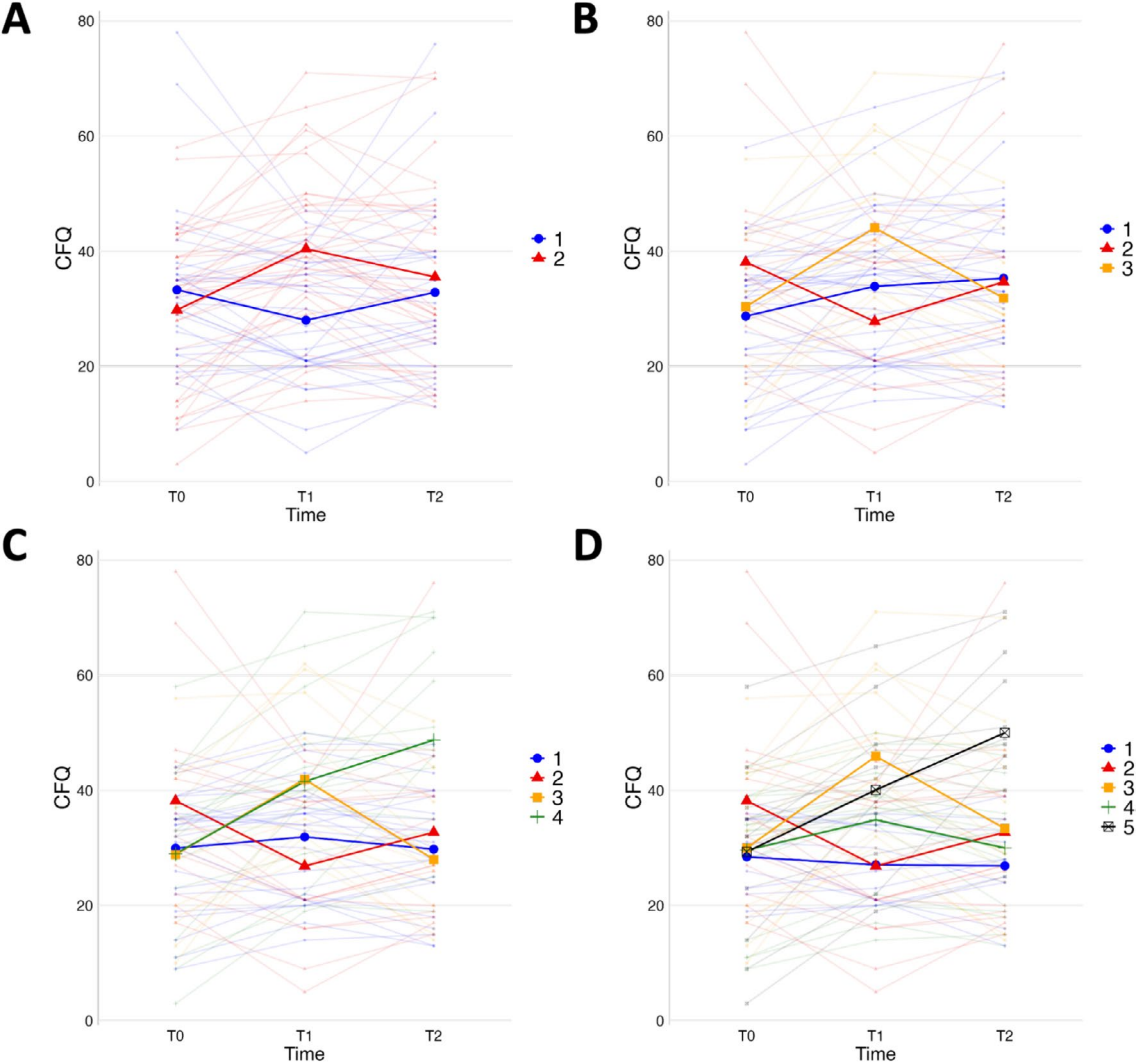
Changes in cognitive complaints between the 3 time‐points for each solution of the PAM algorithm. Individual and mean lines are shown in each plot. The 4‐cluster solution was selected as the optimal clustering. HCs are not shown in the figure. (A) The 2‐cluster solution. (B) The 3‐cluster solution. (C) The 4‐cluster solution. (D) 5‐cluster solution. CFQ, cognitive failure questionnaire; HC, healthy control.

The PAM algorithm was used to identify four partitionings with 2–5 clusters (Table 1). Including healthy controls data, all of them had group sizes larger than 10%. Except for the 5‐cluster solution, each solution revealed distinct trajectories (Figure 2). The 4‐cluster solution scored overall favorable and revealed 4 distinct trajectories. Therefore, this partitioning was identified as the most optimal clustering outcome.

**TABLE 1:** Comparison of the different number of clusters retrieved from the PAM algorithm. Group sizes, average silhouette width, maximum dissimilarity, and average dissimilarity were used to select the optimal solution. Optimal values are indicated in bold. The 4‐cluster solution was identified as the most favorable and used in subsequent analyses.

Number of clusters	Group sizes^a^ (%)	Average silhouette width	Maximum dissimilarity	Average dissimilarity
2	58 (51.78)	**0.35**	34.13	8.59
	54 (48.21)			
3	66 (58.92)	0.33	30.41	7.80
	25 (22.32)			
	21 (18.75)			
4^b^	51 (45.54)	0.32	**29.07**	7.36
	25 (22.32)			
	18 (16.07)			
	18 (16.07)			
5	30 (26.79)	0.28	29.15	**6.45**
	23 (20.54)			
	17 (15.18)			
	29 (25.89)			
	13 (11.61)			

*Note:* Average silhouette width ranges from −1 to 1 and measures the cohesion and separability of the clusters, with higher values indicating more pronounced clusters [64]. Maximal dissimilarity and average dissimilarity are the maximal and average distances of any point to its cluster's medoid, with lower values indicating more coherent clusters.

Abbreviations: HC, healthy control; PAM, partitioning around medoids.

^a^ Including HC, not retained for further analysis.

^b^ The 4‐cluster solution was identified as the optimal clustering.

### 3.3 Cluster Profiles

The four clustered groups had distinct trajectories of cognitive complaints in patients with breast cancer (Figure 2C): (1) a stable trajectory with no changes in cognitive complaints, (2) an improving trajectory with a (non‐significant) higher mean of cognitive complaints followed by a mean decrease between T0 and T1 (*μ* = −11.33, *σ* = 7.35), (3) a short‐term affected trajectory with a mean increase of cognitive complaints between T0 and T1 (*μ* = 13.08, *σ* = 8.68) and recovery between T1 and T2 (*μ* = −13.92, *σ* = 3.82), and (4) a long‐term affected trajectory with a mean increase of cognitive complaints between T0 and T1 (*μ* = 12.53, *σ* = 6.80) and a further increase between T1 and T2 (*μ* = 7.2, *σ* = 8.33). Due to the lack of change in CFQ, the stable group along with baseline were selected as references for the linear mixed models in the subsequent analyses. Profiles of the four groups were compared using demographic and clinical data, shown in Table 2. No significant differences in characteristics were observed between the trajectories.

**TABLE 2:** Characteristics of the whole patient population and the identified groups. No significant differences were observed between the four groups.

Mean (SD) or *n* (%)	Total (*n* = 67)	Stable (*n = 24*)	Improving (*n = 15*)	Short‐term affected (*n = 13*)	Long‐term affected (*n = 15*)	*F*‐test	*p* ^d^
CFQ at baseline	31 (14)	30 (10)	38 (17)	29 (13)	29 (15)	1.658	0.185
Age at baseline (years)	50 (8)	50 (9)	50 (8)	50 (8)	48 (8)	0.188	0.904
BMI at baseline (kg/m^2^)	25 (4)	24 (2)	26 (6)	25 (4)	24 (4)	0.799	0.499
Verbal IQ	110 (8)	110 (6)	113 (9)	108 (6)	108 (12)	0.893	0.450
Education (years)	13 (3)	13 (3)	14 (3)	13 (4)	13 (4)	0.278	0.841
Transition into menopause							
Before diagnosis	27 (40)	10 (42)	10 (67)	3 (23)	4 (27)	/	0.080
Between T0 and T1	20 (30)	6 (25)	2 (13)	4 (31)	8 (53)	/	0.088
Between T1 and T2	6 (9)	3 (13)	0 (0)	3 (23)	0 (0)	/	0.119
Breast cancer stage^a^							
0–1	44 (66)	17 (71)	10 (67)	8 (62)	9 (60)	/	0.912
2	10 (15)	3 (13)	1 (7)	4 (31)	2 (13)	/	0.393
3	13 (20)	4 (17)	4 (27)	1 (8)	4 (27)	/	0.546
Chemotherapy	32 (48)	11 (46)	6 (40)	7 (54)	8 (53)	/	0.845
Additional rounds^b^	10 (31)	5 (45)	2 (33)	1 (14)	2 (25)	/	0.621
Hormone therapy	47 (70)	17 (71)	9 (60)	8 (62)	13 (87)	/	0.351
Tamoxifen	32 (68)	13 (76)	7 (78)	5 (63)	7 (54)	/	0.557
Aromatase inhibitors	15 (32)	4 (31)	2 (22)	3 (38)	6 (46)	/	0.557
Radiotherapy	53 (79)	20 (83)	13 (87)	10 (77)	10 (67)	/	0.547
Targeted therapy^c^	11 (16)	3 (13)	1 (7)	3 (23)	4 (27)	/	0.413

Abbreviations: BMI, body‐mass index; CFQ, cognitive failure questionnaire; IQ, intelligence quotient; SD, standard deviation.

^a^ Breast cancer stage was defined according to the size of the tumor, spread to the lymph nodes, and presence of metastasis (TNM) [43].

^b^ 10 of the patients treated with chemotherapy received additional rounds of one or a combination of the following chemotherapeutic agents: carboplatine 80 mg/m^2^ (*n* = 7), docetaxel 100 mg/m^2^ (*n* = 2), capecitabine 1000 mg/m^2^ (*n* = 4), trastuzumab‐emtansine 3.6 mg/kg (*n* = 2).

^c^ 11 patients received targeted therapy of one or the combination of the following therapies: pembrolizumab (*n* = 1), pertuzumab (*n* = 5), trastuzumab (*n* = 8) or ribociclib (*n* = 2).

^d^ ANOVA or Fisher's exact tests.

### 3.4 Neuropsychological Tests

No significant differences were found between the groups at baseline in any of the neuropsychological tests. Only in WAISd both interaction and within‐group effects were found. No significant effects were observed in ntDom, TMT, AVLTsum, AVLTd, WAISCL, and COWA. Within‐group effects were found in WAISd, AVLTd, WAISCL, and COWA (Supporting Information).

### 3.5 Self‐Reported Measures

No significant differences were found between the groups at baseline in any self‐reported measure, nor was age at diagnosis a significant covariate. An overview of the interaction and within‐group effects is provided in Table S3.

**FIGURE 3:**
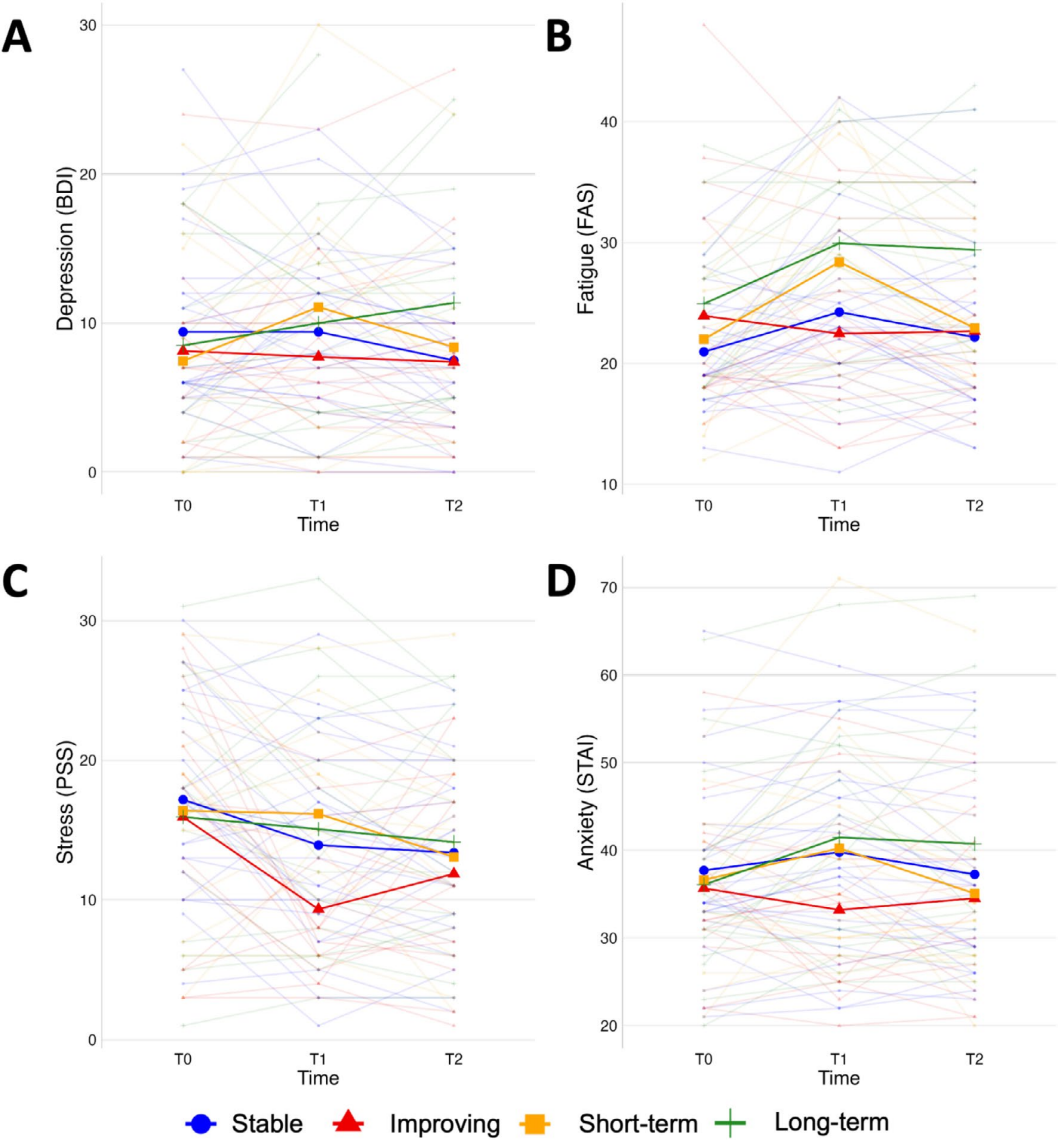
Trajectories of self‐reported measures between the 3 time‐points for each group. Individual and mean lines are shown in each plot. (A) Trajectories of depression, measured by BDI. (B) Trajectories of fatigue, measured by FAS. (C) Trajectories of stress, measured by PSS. (D) Trajectories of anxiety, measured by STAI. BDI, Beck's depression inventory; FAS; fatigue assessment scale; PSS, perceived stress scale; STAI, state–trait anxiety inventory.

Stronger increases in depression (Figure 3A) were observed at T1 in the short‐term affected group (*β* = 3.615, *p =* 0.030) and at T2 in the long‐term affected group (*β* = 5.119, *p =* 0.002) compared to baseline and the stable group. Within‐group analysis revealed a significant increase at T1 in the short‐term affected group (*β* = 3.615, *p =* 0.007) and at T2 in the long‐term affected group (*β* = 3.203, *p =* 0.015).

A stronger decrease in fatigue (Figure 3B) was observed at T1 in the improving group (*β* = −4.758, *p =* 0.009) compared to baseline and the stable group. Within‐group analysis did not reveal decreases in the improving group, but an increase at T1 in the stable group (*β* = 3.292, *p =* 0.004), in the short‐term affected group (*β* = 6385, *p <* 0.001), and in the long‐term affected group both at T1 (*β* = 5.000, *p <* 0.001) and T2 (*β* = 4.467, *p =* 0.002).

No differences between the groups were observed for stress (Figure 3C). However, within‐group analysis revealed a significant decrease at T1 in the stable group (*β* = −3.113, *p =* 0.010) and in the improving group (*β* = −6.600, *p* < 0.001). At T2 a significant decrease was observed in the stable group (*β* = −3.655, *p =* 0.003), the improving group (*β* = −4.067, *p =* 0.007), and the short‐term affected group (*β* = −3.308, *p =* 0.041).

A stronger increase in anxiety (Figure 3D) was observed at T2 in the long‐term affected group (*β* = 5.125, *p =* 0.029) compared to baseline and the stable group. Within‐group analysis revealed a significant increase in the long‐term affected group both at T1 (*β* = 5.400, *p =* 0.004) and at T2 (*β* = 4.667, *p =* 0.011).

### 3.6 Serum Markers

No significant differences at baseline were observed between the groups for any of the serum markers. Significant interaction and/or within‐group effects were found only for sTREM2, BDNF, and MCP‐1, but not for the other markers. Specifically, sTREM2 showed both significant interaction and within‐group effects. MCP‐1 exhibited significant within‐group effects, but no interaction effects. In contrast, BDNF showed significant interaction effects, without significant within‐group changes. A detailed overview can be found in the Supporting Information.

### 3.7 Magnetic Resonance Imaging

**FIGURE 4:**
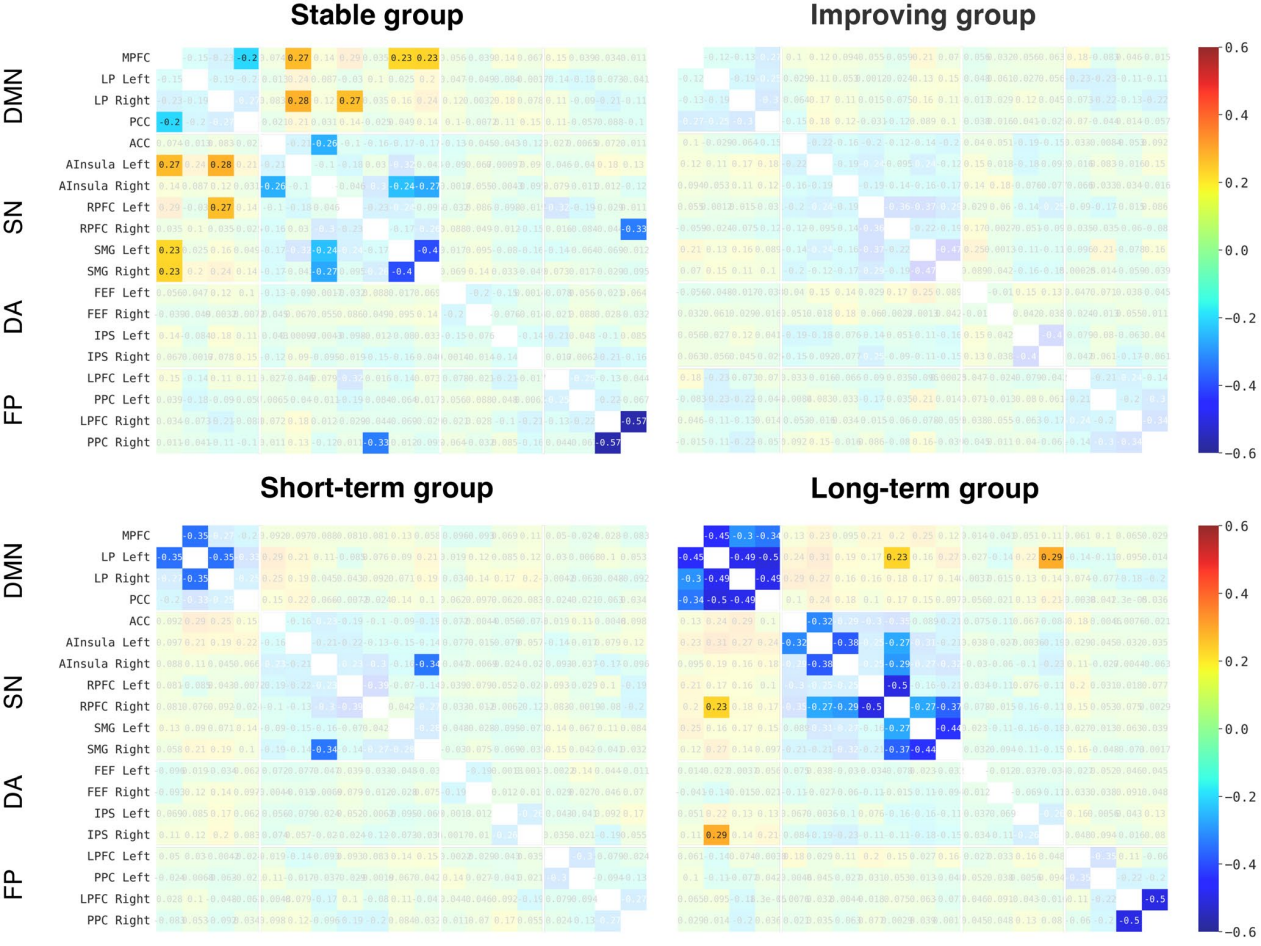
Heat maps of the change in functional connectivity patterns between T0 and T1 for each group. The change in correlation coefficients of each pair of regions is shown. Connections that were not significant after FDR correction are made transparent. All groups, except the improving group, showed significant changes in functional connectivity between T0 and T1, but not between T1 and T2. A list of regions and their abbreviations is provided in Table S2. DMN, default mode network; DA, dorsal attention network; FDR, false discovery rate; FP, frontoparietal network; SN, salience network.

In the ROI‐to‐ROI analysis (Figure 4), no significant differences in connectivity were found between the groups in any of the 4 networks. Within‐group analysis revealed that, except for the improving group, all other groups showed significant decreases in connections within the default mode network and salience network from T0 to T1 (thresholded at *p* < 0.05, FDR‐corrected). In addition, the stable group and the long‐term affected group showed significant decreases in the fronto‐parietal network. No significant changes were observed between T1 and T2.

Using graph theory analysis, no baseline differences in the graph metrics were found between the groups. No differences between the groups or within‐group effects were observed in global efficiency, clustering coefficient, and characteristic path length. A stronger increase in local efficiency was observed at T2 in the long‐term affected group (*β* = 0.012, *β*_standardized_ = 0.012, SE = 0.006, 95% CI = [0.001, 0.023], *p =* 0.041) compared to baseline and the stable group. However, within‐group analysis did not reveal any significant changes.

## 4 Discussion

Current research on CRCI frequently overlooks the variability of cognitive problems experienced by breast cancer patients, potentially leading to suboptimal treatment strategies. This study addresses this gap by categorizing patients into different groups according to their cognitive complaints over time, while incorporating clinical, demographic, and neurobiological data. The findings confirm the heterogeneity in cognitive complaints and offer valuable insights into its contributing factors.

Within the patients cohort, the stable and improving group, containing 58% of patients, did not show worsening in cognitive complaints after diagnosis. On the other hand, the short‐term group, containing 19% of patients, endured worse cognitive complaints at T1 but was able to recover afterwards. Unfortunately, the long‐term group, containing 22% of patients, did not show such recovery, with cognitive complaints persisting for more than a year after diagnosis. Consequently, the clustering algorithm revealed strong variability over time in patients with cognitive complaints, which would have been overlooked using a single patient group or treatment‐based groups.

The worsening of cognitive complaints in these two poorer groups was strongly associated with higher levels of anxiety, fatigue, depression, and stress, which persisted in the long‐term group. This suggests that these factors are strongly associated with both susceptibility to and recovery from cognitive complaints among patients with breast cancer. Previous cross‐sectional and longitudinal studies have also revealed associations between subjective cognitive decline and fatigue, depression, anxiety, and stress [15, 65, 66, 67, 68]. Fatigue and distress are well‐described side effects of cancer and treatment. It has been suggested that they both play a pivotal role in CRCI [69, 70]. Although not assessed within this study, other psychosocial factors including adverse childhood events and social coherence have been associated with subjective cognitive decline in patients with breast cancer, along with fatigue, depression, and stress [71, 72]. Interventions focused on improving these psychosocial conditions can potentially decrease cognitive complaints and increase the quality of life after cancer and treatment [73]. For instance, mindfulness‐based interventions have shown promising results in decreasing cognitive complaints, along with improving stress and anxiety levels [74, 75, 76].

Interestingly, the cluster profiles did not significantly differ in any of the clinical characteristics, including chemotherapy. Yet, chemotherapy has been reported as a major risk factor for both objective and subjective cognitive decline [7, 9, 10, 77], although strong variations in the severity and duration of these cognitive complaints have been shown among chemotherapy‐treated patients [78]. One potential side effect of chemotherapy is transitioning into menopause [79], which may be temporary for some patients but persistent for others [80]. Importantly, increased cognitive complaints are common during and after menopausal transition, with treatment‐induced menopause potentially causing more severe complaints than naturally induced menopause [81]. In this study, although not significant, there were slightly more patients undergoing menopause during treatment among the poorer cognitive groups. Larger population‐based studies are necessary to investigate whether menopausal transition contributes to the heterogeneity in cognitive decline among chemotherapy‐treated patients.

No clear associations between serum markers and the cognitive groups were found. This might be due to the similar number of chemotherapy‐treated patients across groups, as alterations are mostly reported during and after chemotherapy. Moreover, studies report more consistent associations with objective cognition rather than subjective cognition [30]. However, among chemotherapy‐treated patients specifically, these markers may still account for variability in subjective cognitive complaints. In fact, a negative association has been established between the inflammatory marker MCP‐1 and subjective cognition within chemotherapy‐treated patients [82]. Besides, recent studies have identified several other markers that are predictive of CRCI regardless of treatment. C‐reactive protein (CRP) levels at diagnosis have been shown to predict both subjective and objective long‐term cognitive impairment [83, 84]. Apolipoprotein E4 positivity may also serve as a predictor of CRCI, indicating a potential genetic risk factor [85, 86]. Therefore, while some serum markers may primarily reflect indirect neurotoxic effects of chemotherapy, others appear more directly predictive of CRCI. Further research is necessary to establish such markers of CRCI heterogeneity.

In both rs‐fMRI analyses, no significant differences between the groups were found. However, in the ROI‐to‐ROI analysis, significant decreases in connectivity within regions of the default mode and salience network were observed between T0 and T1 in all groups except the improving group, which has lower cognitive complaints and psychosocial factors. These findings align with prior research in patients with breast cancer, which has associated decreased connectivity in the default mode and salience networks with subjective cognitive decline, anxiety, and depression [21, 87, 88, 89]. Interestingly, the long‐term group showed the most significant decreased connections at T1, suggesting early disruptions might predict persistent cognitive complaints. Nevertheless, the role of functional connectivity in CRCI heterogeneity needs further investigation.

It is important to acknowledge the limitations of the current study, as addressing them in future research could enhance findings. First of all, concerns have been raised about the reliability of self‐reported measures of cognition. Response biases, where individuals report fewer complaints than their genuine experiences, and state‐dependent biases, where responses are related to an individual's current emotional state, have been reported [90, 91]. Additionally, personality traits can influence these results, with less confident and more introverted individuals tending to score worse. To improve the reliability of patient‐reported cognitive outcomes, incorporating their partner's observations has been suggested [92]. Alternatively, neuropsychological tests could be used as the outcome measure for the clustering algorithm. However, as observed in this study, associations between objective and self‐reported cognitive decline are often missing in CRCI. Patients experiencing cognitive complaints often still perform within normal ranges of the tests [93, 94]. A potential explanation is that the tests may lack sensitivity to CRCI, while the controlled testing environment and compensatory brain mechanisms could also contribute to why most patients continue to perform well [95, 96]. Objective cognitive decline could also be assessed using discrepancy scores, which measure the difference between two related neuropsychological tests and may better align with self‐reported cognitive decline [97]. Secondly, unsupervised clustering methods like the PAM algorithm provide an approximation of the underlying heterogeneity within the cohort, but validation in other cohorts is necessary [37, 98]. Larger cohorts and more time points could also improve the reliability of the PAM algorithm. Finally, this study had a limited sample size in each group, which may reduce statistical power and increase the risk of type II errors. Standardized coefficients suggested large effect sizes for the self‐reported measures (0.4–0.8) despite the small sample size, though smaller effects may have gone undetected. Future studies with larger samples are needed to both confirm the observed effects and provide certainty regarding smaller or non‐significant relationships.

## 5 Conclusion

This study highlights the heterogeneity in cognitive complaints among patients with breast cancer. Within this cohort, four distinct trajectories of cognitive complaints were identified, which were mainly differentiated by psychosocial measures, rather than neuropsychological tests, serum markers, or functional connectivity. Further research is needed to examine this heterogeneity in order to identify potential predictive markers of cognitive decline. This study underscores the influence of psychosocial factors on self‐reported cognition. Interventions focused on decreasing stress, anxiety, depression, and fatigue could significantly decrease cognitive complaints, resulting in improved quality of life during and after (breast) cancer treatment.

## Author Contributions

**Rob Colaes:** conceptualization (equal), formal analysis (lead), methodology (lead), writing – original draft (lead), writing – review and editing (equal). **Gwen Schroyen:** conceptualization (equal), investigation (equal), project administration (equal), writing – review and editing (equal). **Rebeca Alejandra Gavrila Laic:** writing – review and editing (equal). **Jeroen Blommaert:** conceptualization (equal), writing – review and editing (equal). **Sigrid Hatse:** conceptualization (equal), resources (equal), writing – review and editing (equal). **Ann Smeets:** conceptualization (equal), investigation (equal), resources (equal), writing – review and editing (equal). **Stefan Sunaert:** conceptualization (equal), formal analysis (equal), funding acquisition (equal), investigation (equal), supervision (equal), writing – review and editing (equal). **Sabine Deprez:** conceptualization (equal), funding acquisition (equal), investigation (equal), project administration (equal), supervision (equal), writing – review and editing (equal).

## Conflicts of Interest

The authors declare no conflicts of interest.

## Supporting information


**Data S1:** cam471130‐sup‐0001‐DataS1.docx.

## Data Availability

Data will be made available upon reasonable request.
