# Supplementary material for: Trajectories of Cognitive Complaints in Patients With Breast Cancer and Their Association With Psychosocial and Neurobiological Factors
Source: Cancer Med. 2025 Aug 7;14(15):e71130. doi: 10.1002/cam4.71130 (PMC12329573; doi:10.1002/cam4.71130)
Supplement: Supplementary file 1 — Data S1: cam471130‐sup‐0001‐DataS1.docx. [file CAM4-14-e71130-s001.docx]

## Methods

### Healthy controls

Supplementary table 1: Characteristics of the 45 healthy women included in this study.

| **Characteristic** | **Mean** | **SD** | **Range** |
| --- | --- | --- | --- |
| CFQ at baseline | 26 | 10 | [8, 58] |
| Age at baseline  (years) | 46 | 11 | [26, 64] |
| BMI at baseline  (kg/m^2^) | 23 | 4 | [18, 34] |
| Verbal IQ | 108 | 8 | [90, 127] |
| Education  (years) | 15 | 2 | [6, 17] |

*Abbreviations: HC, healthy control; CFQ, cognitive failure questionnaire; BMI, body-mass index; IQ, intelligence quotient; SD, standard deviation.*

### Serum markers

Serum samples were collected at each time-point for all participants in 4-mL BD Vacutainer SST II Advance tubes and were centrifuged (1600xg for 15 min at 4°C), aliquoted and stored at -80 °C until analysis. Cytometric bead arrays were run in duplicate on separate plates, with a standard curve included on each plate. Mean values across duplicates (separate plate assays) were used for analysis. Values above and below the detection limits were converted to the respective detection limit. This was the case for 18 data points of BDNF. The markers were analyzed in two batches. Twenty-two participants had one or two time points analyzed in both batches. If this was the case, values were retained from the batch that included all three time-points so that all the patient’s samples were analyzed in the same run. For intra- and interplate variability, the coefficient of variation (CV) was calculated based on the two internal controls measured on every plate. Three markers, VILIP-1, TGF-β, and β-NGF, had a CV for interplate variability higher than 30% for at least one run and were consequently excluded from the analysis as recommended in the LEGENDplex^TM^ manual.

For the NfL, mean values across triplicates were used for analysis. NfL was also analyzed in two sessions and the values of the session that included all three time-points were retained. The intra- and interplate variability was 22% and 45% respectively for the first session and 16% and 42% for the second session.

### Magnetic resonance imaging

All images were converted to the Brain Imaging Data Structure (BIDS) [^99^](https://paperpile.com/c/W6ksH6/pvGLU). All images were checked for abnormalities and artifacts. Subsequently, quantitative quality measurements for the Blood Oxygenation-Level Dependent (BOLD) images were computed using MRIQC version 22.0.1 [^100^](https://paperpile.com/c/W6ksH6/FChHR). Images with an average framewise displacement > 0.4 were checked by a neuroradiologist (Prof. Dr. Stefan Sunaert) [^101^](https://paperpile.com/c/W6ksH6/fJ43e).

Preprocessing was done using fMRIprep version 20.2.7 [^102^](https://paperpile.com/c/W6ksH6/VG18o), which included susceptibility distortion correction [^103^](https://paperpile.com/c/W6ksH6/fXh6j), slice-time correction, re-alignment, co-registration, spatial normalization to the MNI152NLin2009cAsym and the MNI152NLin6Asym. Global signals of WM and CSF were calculated. Automatic removal of motion artifacts using independent component analysis (ICA-AROMA) was performed to identify motion and noise components [^104^](https://paperpile.com/c/W6ksH6/Eh4cv). Additionally, a T1w-reference map was computed after registration of 3 T1w images using FreeSurfer 6.0.1 [^105^](https://paperpile.com/c/W6ksH6/NJczq). The BOLD images were then denoised using denoiser version 1.0.1 [^106^](https://paperpile.com/c/W6ksH6/tEmaN). A bandpass filter was used (0.09Hz - 0.1Hz) and the following nuisance terms identified by fMRIprep were used as regressors: CSF and WM signal, realignment (6 parameters), and ICA-AROMA motion confounds (variable number of parameters).

For the ROI-to-ROI analysis, the images were analyzed in the CONN (v22a) functional connectivity toolbox [^107^](https://paperpile.com/c/W6ksH6/FPKBW) (implemented in MATLAB R2022a). Additional smoothing was done (FWHM=6 mm). Regions of interest (ROIs) of attention-, emotion-, and executive function-related networks were selected from the CONN network atlas. This resulted in a total of 19 regions with 171 connections (supplementary table 2). Functional connectivity strength was represented by Fisher-transformed bivariate correlation coefficients for each pair of ROIs. Cluster-level inferences were based on parametric statistics within- and between- each pair of networks, with networks identified using a complete-linkage hierarchical clustering procedure based on ROI-to-ROI anatomical proximity and functional similarity metrics [^108^](https://paperpile.com/c/W6ksH6/kelyH). Results were thresholded using a false-discovery rate corrected (p-FDR<0.05) connection-level threshold. Connection matrices derived from CONN were visualized using heat maps, constructed in python (v3.11.5). For this part of the analysis, we exclusively considered participants who had resting-state fMRI scans at all three designated time-points as CONN cannot handle missing data.

Subsequently, graph theory was applied to the denoised images. First, cortical parcellation with the Desikan-Killiany-Tourville atlas (DKT) was performed on the T1w-reference image using FastSurfer version 2.0.4 [^109^](https://paperpile.com/c/W6ksH6/PWlUg) [^110^](https://paperpile.com/c/W6ksH6/rKmIQ) [^111^](https://paperpile.com/c/W6ksH6/kAwDl). The connectome lookup table was converted using MRtrix version 3.0.4 [^112^](https://paperpile.com/c/W6ksH6/lTp23). The parcellated image with 78 ROIs was transformed to subject-specific space of the BOLD image using ANTs version 2.3.5 [^113^](https://paperpile.com/c/W6ksH6/UhiW4). The average time-series in each of these ROIs was derived from the BOLD image using FSL version 6.0.5.1 [^114^](https://paperpile.com/c/W6ksH6/GaTiG). Graph theory analysis was performed using in-house developed MATLAB (r2022a) scripts and the Brain Connectivity toolbox version 2019-03-03 [^115^](https://paperpile.com/c/W6ksH6/PFLXM). Weighted connectomes were constructed using the partial correlations between the average time-series and self-connections were removed. Whole-brain graph theory measures of characteristic path length, clustering coefficient, and global and local efficiency were derived for this analysis. Characteristic path length and global efficiency were calculated using Dijkstra’s algorithm, with the connection-length matrix defined by the inverse edge weights (with self-connections having zero-length). Clustering coefficient and local efficiency measures were calculated as recommended by Wang et al. [^116^](https://paperpile.com/c/W6ksH6/nzSTK). For each connectome, 1000 random graphs were constructed through random edge permutation, excluding graphs with disconnected nodes. Normalized graph measures were calculated by dividing the original graph measures of the connectome by the median of the graph measures of equivalent graphs. Contrary to the ROI-to-ROI, participants with missing rs-fMRI were included.

Supplementary table 2: List of regions of the 4 networks used for the ROI-to-ROI analysis. In total 19 regions were included from the CONN network atlas.

| **Region of interest** | **MNI-coordinates** | **Number of voxels** |
| --- | --- | --- |
| *Default mode network* | | |
| Medial prefrontal cortex (MPFC) | (1, 55, -3) | 1346 |
| Left lateral parietal cortex (LP Left) | (-39, -77, 33) | 1041 |
| Right lateral parietal (LP Right) | (47, -67, 29) | 1326 |
| Posterior cingulate cortex (PCC) | (1, -61, 38) | 4833 |
| *Salience network* | | |
| Anterior cingulate cortex (ACC) | (0, 22, 35) | 1063 |
| Left anterior insula (AInsula Left) | (-44, 13, 1) | 446 |
| Right anterior insula (AInsula Right) | (47, 14, 0) | 388 |
| Left rostral prefrontal cortex  (RPFC Left) | (-32, 45, 27) | 1166 |
| Right rostral prefrontal cortex  (RPFC Right) | (32, 46, 27) | 581 |
| Left supramarginal gyrus  (SMG Left) | (-60, -39, 31) | 233 |
| Right supramarginal gyrus  (SMG Right) | (62, -35, 32) | 284 |
| *Dorsal attention network* | | |
| Left frontal eye field (FEF Left) | (-27, -9, 64) | 88 |
| Right frontal eye field (FEF Right) | (30, -6, 64) | 54 |
| Left intraparietal sulcus (IPS Left) | (-39, -43, 52) | 3285 |
| Right intraparietal sulcus (IPS Right) | (39, -42, 54) | 3137 |
| *Fronto-parietal network* | | |
| Left lateral prefrontal cortex  (LPFC Left) | (-43, 33, 28) | 1703 |
| Left posterior parietal cortex  (PPC Left) | (-46, -58, 49) | 832 |
| Right lateral prefrontal cortex  (LPFC Right) | (41, 38, 30) | 1758 |
| Right posterior parietal cortex  (PPC Right) | (52, -52, 45) | 837 |

## Supplementary - results

### Self-reported measures

Supplementary table 3: Results from the linear mixed effects models and post-hoc within-group analysis for the four self-reported measures: BDI, FAS, PSS, and STAI. T0 and the stable group were used as the reference for the linear mixed effects model.

|  | **Coefficient** | **Standardized coefficient** | **SE** | ***p-value*** | **95% CI** |
| --- | --- | --- | --- | --- | --- |
| **BDI** | | | | | |
| *Time-by-group interaction effects* | | | | | |
| Intercept | 14.590 | 0.090 | 4.692 | **0.003** | [5.620, 23.564] |
| Age at diagnosis | -0.104 | -0.130 | 0.091 | 0.255 | [-0.278, 0.069] |
| T1 x improving | -0.400 | -0.062 | 1.577 | 0.800 | [-3.418, 2.618] |
| T2 x improving | 1.183 | 0.185 | 1.577 | 0.454 | [-1.835, 4.201] |
| T1 x short-term | 3.615 | 0.564 | 1.650 | **0.030** | [0.458, 6.773] |
| T2 x short-term | 2.840 | 0.443 | 1.650 | 0.088 | [-0.318, 5.997] |
| T1 x long-term | 1.025 | 0.160 | 1.601 | 0.523 | [-2.036, 4.094] |
| T2 x long-term | 5.119 | 0.798 | 1.627 | **0.002** | [2.003, 8.230] |
| *Within-group effects* | | | | | |
| Intercept | 14.590 | 0.90 | 4.692 | **0.003** | [5.620, 23.564] |
| T1 x stable | 0.000 | 0.000 | 0.978 | 1.000 | [-1.872, 1.872] |
| T2 x stable | 1.917 | -0.299 | 0.978 | 0.052 | [-3.788, -0.045] |
| T1 x improving | -0.400 | -0.062 | 1.237 | 0.747 | [-2.767, 1.967] |
| T2 x improving | -0.733 | -0.114 | 1.237 | 0.554 | [-3.101, 1.634] |
| T1 x short-term | 3.615 | 0.564 | 1.329 | **0.007** | [1.072, 6.158] |
| T2 x short-term | 0.923 | 0.143 | 1.329 | 0.489 | [-1.620, 3.466] |
| T1 x long-term | 1.025 | 0.160 | 1.268 | 0.420 | [-1.398, 3.456] |
| T2 x long-term | 3.203 | 0.499 | 1.300 | **0.015** | [0.712, 5.688] |
| **FAS** | | | | | |
| *Time-by-group interaction effects* | | | | | |
| Intercept | 24.364 | -0.441 | 5.040 | **< 0.001** | [14.727, 34.001] |
| Age at diagnosis | -0.069 | -0.073 | 0.097 | 0.483 | [-0.255, 0.118] |
| T1 x improving | -4.758 | -0.641 | 1.788 | **0.009** | [-8.181, -1.335] |
| T2 x improving | -2.475 | -0.333 | 1.788 | 0.169 | [-5.898, 0.948] |
| T1 x short-term | 3.093 | 0.417 | 1.871 | 0.101 | [-0.488, 6.674] |
| T2 x short-term | -0.285 | -0.038 | 1.871 | 0.879 | [-3.867, 3.296] |
| T1 x long-term | 1.708 | 0.230 | 1.788 | 0.341 | [-1.715, 5.131] |
| T2 x long-term | 3.258 | 0.439 | 1.788 | 0.071 | [-0.165, 6.681] |
| *Within-group effects* | | | | | |
| Intercept | 24.364 | -0.441 | 5.040 | **< 0.001** | [14.727, 34.001] |
| T1 x stable | 3.292 | 0.444 | 1.109 | **0.004** | [1.1689, 5.415] |
| T2 x stable | 1.208 | 0.163 | 1.109 | 0.278 | [-0.915, 3.331] |
| T1 x improving | -1.467 | -0.198 | 1.403 | 0.298 | [-4.152, 1.219] |
| T2 x improving | -1.267 | -0.171 | 1.403 | 0.368 | [-3.952, 1.419] |
| T1 x short-term | 6.385 | 0.860 | 1.507 | **< 0.001** | [3.500, 9.269] |
| T2 x short-term | 0.923 | 0.124 | 1.507 | 0.541 | [-1.961, 3.807] |
| T1 x long-term | 5.000 | 0.674 | 1.403 | **< 0.001** | [2.315, 7.685] |
| T2 x long-term | 4.467 | 0.602 | 1.403 | **0.002** | [1.781, 7.152] |
| **PSS** | | | | | |
| *Time-by-group interaction effects* | | | | | |
| Intercept | 19.701 | 0.348 | 5.394 | **< 0.001** | [9.385, 30.0114] |
| Age at diagnosis | -0.054 | -0.057 | 0.104 | 0.606 | [-0.253, 0.145] |
| T1 x improving | -3.487 | -0.460 | 1.912 | 0.071 | [-7.144, 0.174] |
| T2 x improving | -0.412 | -0.054 | 1.912 | 0.830 | [-4.069, 3.249] |
| T1 x short-term | 2.882 | 0.380 | 1.999 | 0.152 | [-0.942, 6.710] |
| T2 x short-term | 0.347 | 0.046 | 1.999 | 0.862 | [-3.477, 4.175] |
| T1 x long-term | 2.247 | 0.296 | 1.912 | 0.242 | [-1.411, 5.907] |
| T2 x long-term | 1.855 | 0.244 | 1.912 | 0.334 | [-1.802, 5.515] |
| *Within-group effects* | | | | | |
| Intercept | 19.701 | 0.348 | 5.394 | **< 0.001** | [9.385, 30.0114] |
| T1 x stable | -3.113 | -0.410 | 1.197 | **0.010** | [-5.405, -0.824] |
| T2 x stable | -3.655 | -0.482 | 1.197 | **0.003** | [-5.947, -1.366] |
| T1 x improving | -6.600 | -0.870 | 1.491 | **< 0.001** | [-9.453, -3.747] |
| T2 x improving | -4.067 | -0.536 | 1.491 | **0.007** | [-6.920, -1.213] |
| T1 x short-term | -0.231 | -0.030 | 1.601 | 0.886 | [-3.296, 2.834] |
| T2 x short-term | -3.308 | -0.436 | 1.601 | **0.041** | [-6.373, -0.243] |
| T1 x long-term | -0.867 | -0.114 | 1.491 | 0.562 | [-3.720, 1.987] |
| T2 x long-term | -1.800 | -0.237 | 1.491 | 0.230 | [-4.653, 1.053] |
| **STAI** | | | | | |
| *Time-by-group interaction effects* | | | | | |
| Intercept | 38.978 | 0.020 | 8.409 | **< 0.001** | [22.896, 55.060] |
| Age at diagnosis | -0.026 | -0.018 | 0.163 | 0.876 | [-0.337, 0.286] |
| T1 x improving | -4.55 | -0.406 | 2.314 | 0.051 | [-8.979, -0.121] |
| T2 x improving | -0.675 | -0.060 | 2.314 | 0.771 | [-5.104, 3.754] |
| T1 x short-term | 1.532 | 0.137 | 2.421 | 0.528 | [-3.102, 6.166] |
| T2 x short-term | -1.080 | -0.096 | 2.421 | 0.656 | [-5.714, 3.553] |
| T1 x long-term | 3.317 | 0.296 | 2.314 | 0.154 | [-1.112, 7.745] |
| T2 x long-term | 5.125 | 0.457 | 2.314 | **0.029** | [0.696, 9.554] |
| *Within-group effects* | | | | | |
| Intercept | 38.978 | 0.020 | 8.409 | **< 0.001** | [22.896, 55.060] |
| T1 x stable | 2.083 | 0.186 | 1.435 | 0.149 | [-0.663, 4.830] |
| T2 x stable | -0.458 | -0.041 | 1.435 | 0.750 | [-3.205, 2.288] |
| T1 x improving | -2.467 | -0.210 | 1.815 | 0.177 | [-5.941, 1.008] |
| T2 x improving | -1.133 | -0.101 | 1.815 | 0.533 | [-4.608, 2.341] |
| T1 x short-term | 3.615 | 0.322 | 1.950 | 0.066 | [-0.117, 7.347] |
| T2 x short-term | -1.538 | -0.137 | 1.950 | 0.432 | [-5.270, 2.193] |
| T1 x long-term | 5.400 | 0.481 | 1.815 | **0.004** | [1.926, 8.874] |
| T2 x long-term | 4.667 | 0.416 | 1.815 | **0.011** | [1.192, 8.141] |

*Abbreviations: BDI, Beck’s depression inventory; FAS; fatigue assessment scale; PSS, perceived stress scale; STAI, state-trait anxiety inventory.*

### Neuropsychological tests

No significant differences were found between the groups at baseline in any of the neuropsychological tests. Age was a significant covariate in ntDom, WAISCL, WAISd, AVLTsum, and AVLTd. No differences between the groups or within-group effects were observed in ntDom, TMT, and AVLTsum.

Stronger increases in WAISd (supplementary figure 1A) were observed at T2 in the improving group (*β*=0.875, *β_standardized_*=0.801, SE=0.373, 95%CI=[0.161, 1.589], *p=*0.021) compared to baseline and the stable group. Within-group analysis revealed a significant increase at T2 in the improving group (*β*=1.000, *β_standardized_*=0.915, SE=0.293, 95%CI=[0.440, 1.560], *p<*0.001).

No differences between the groups were observed in AVLTd (supplementary figure 1B). However, within-group analysis revealed a significant decrease at T2 in the long-term affected group (*β*=-1.200, *β_standardized_*=-0.436, SE=0.545, 95%CI=[-2.243, -0.157], *p=*0.029).

No differences between the groups were observed in WAISCL (supplementary figure 1C). However, within-group analysis revealed a significant increase at T2 in the long-term affected group (*β*=1.000, *β_standardized_*=0.835, SE=0.326, 95%CI=[0.375, 1.625], *p=*0.003).

No differences between the groups were observed in COWA (supplementary figure 1D). However, within-group analysis revealed a significant increase at T2 in the improving group (*β*=5.867, *β_standardized_*=0.461, SE=1.854, 95%CI=[2.318, 9.415], *p=*0.002).


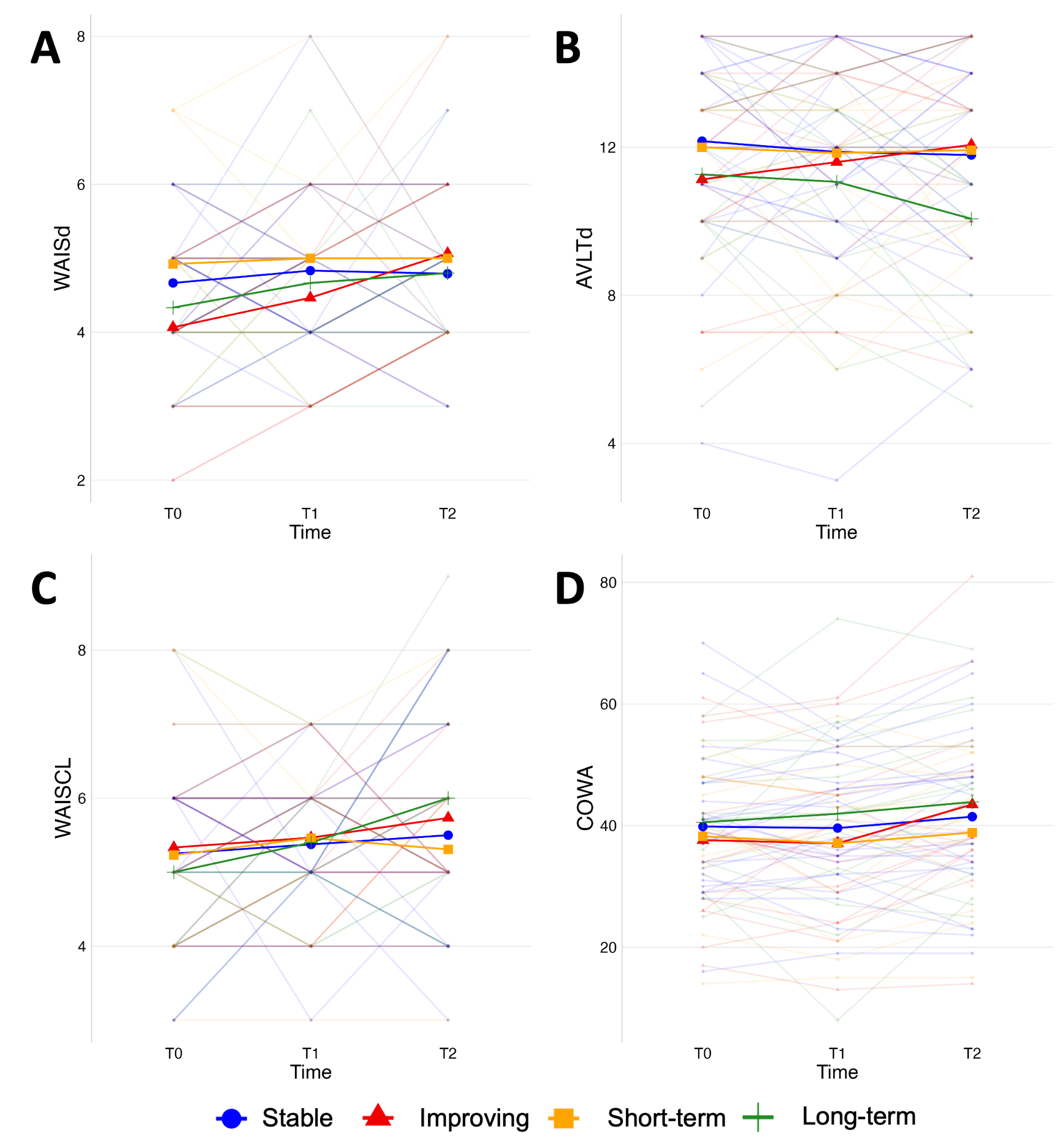


Supplementary figure 1: Changes in neuropsychological tests between the 3 time-points for each group. Individual and mean lines are shown in each plot. A) Trajectories of WAISd. B) Trajectories of AVLTd. C) Trajectories of WAISCL. D) Trajectories of COWA. Abbreviations: WAISCL, Wechsler adult intelligence scale III digit symbol; AVLTd, auditory verbal learning test delayed recall; WAISd, Wechsler adult intelligence scale III digit span backwards; COWA, controlled oral word association test.

### Serum markers

No significant differences at baseline were found between the groups in any of the serum markers. The number of storage days was in none of the markers a significant covariate. No differences between the groups of within-group effects were observed for VEG-F, IL-6, sTREM-1, β-NGF, TNF-ɑ, sRAGE, CXC3CL1, and NfL.

No differences between the groups were observed for MCP-1 (supplementary figure 2A). However, within-group analysis revealed a significant decrease at T2 in the stable group (*β*=-225.800, *β_standardized_*=-0.787, SE=113.904, 95%CI=[-442.670, -8.596], *p=*0.049).

A stronger decrease in sTREM2 (supplementary figure 2B) was observed at T1 in the short-term affected group (*β*=-3206.045, *β_standardized_*=-0.786, SE=1400.493, 95%CI=[-5884.398, -528.282], *p=*0.024) compared to baseline and the stable group. Within-group analysis revealed a significant decrease at T1 in the short-term affected group (*β*=-2974.021, *β_standardized_*=-0.729, SE=1331.917, 95%CI=[-5510.563, -429.758], *p=*0.027).

A smaller decrease in BDNF (supplementary figure 2C) was observed at T2 in the long-term group (*β*=9207.251, *β_standardized_*=0.645, SE=4584.378, 95%CI=[439.641, 17970.746], *p=*0.047) compared to baseline and the stable group. However, within-group analysis did not reveal any significant changes.


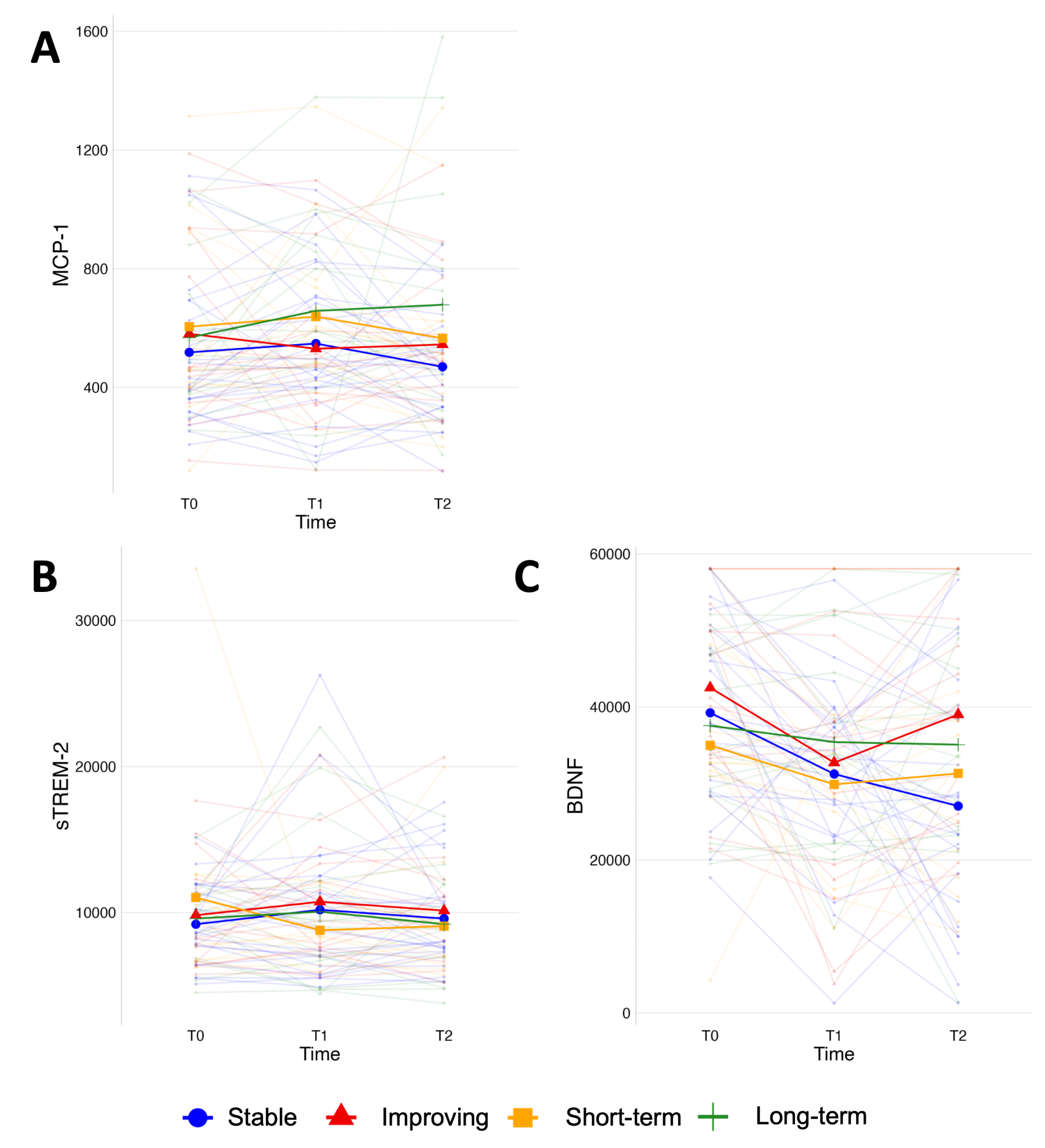


Supplementary figure 2: Changes in serum markers between the 3 time-points for each group. Individual and mean lines are shown in each plot. A) Trajectories of MCP-1. B) Trajectories of sTREM-2. C) Trajectories of BDNF. Abbreviations: MCP-1, monocyte chemoattractant protein-1 (MCP-1); sTREM-1, soluble triggering receptor expressed on myeloid cells 1; BDNF, brain-derived neurotrophic factor.

References

99. [Gorgolewski KJ, Auer T, Calhoun VD, et al. The brain imaging data structure, a format for organizing and describing outputs of neuroimaging experiments. *Sci Data*. 2016;3:160044.](http://paperpile.com/b/W6ksH6/pvGLU)

100. [Esteban O, Birman D, Schaer M, Koyejo OO, Poldrack RA, Gorgolewski KJ. MRIQC: Advancing the automatic prediction of image quality in MRI from unseen sites. *PLoS One*. 2017;12(9):e0184661.](http://paperpile.com/b/W6ksH6/FChHR)

101. [Jenkinson M, Bannister P, Brady M, Smith S. Improved optimization for the robust and accurate linear registration and motion correction of brain images. *Neuroimage*. 2002;17(2):825-841.](http://paperpile.com/b/W6ksH6/fJ43e)

102. [Esteban O, Markiewicz CJ, Blair RW, et al. fMRIPrep: a robust preprocessing pipeline for functional MRI. *Nat Methods*. 2019;16(1):111-116.](http://paperpile.com/b/W6ksH6/VG18o)

103. [Wang S, Peterson DJ, Gatenby JC, Li W, Grabowski TJ, Madhyastha TM. Evaluation of Field Map and Nonlinear Registration Methods for Correction of Susceptibility Artifacts in Diffusion MRI. *Front Neuroinform*. 2017;11:17.](http://paperpile.com/b/W6ksH6/fXh6j)

104. [Pruim RHR, Mennes M, van Rooij D, Llera A, Buitelaar JK, Beckmann CF. ICA-AROMA: A robust ICA-based strategy for removing motion artifacts from fMRI data. *Neuroimage*. 2015;112:267-277.](http://paperpile.com/b/W6ksH6/Eh4cv)

105. [Reuter M, Rosas HD, Fischl B. Highly accurate inverse consistent registration: a robust approach. *Neuroimage*. 2010;53(4):1181-1196.](http://paperpile.com/b/W6ksH6/NJczq)

106. [Tambini A, Gorgolewski KJ. *Denoiser: A Nuisance Regression Tool for fMRI BOLD Data*. Zenodo; 2020. doi:](http://paperpile.com/b/W6ksH6/tEmaN)[10.5281/ZENODO.4033939](http://dx.doi.org/10.5281/ZENODO.4033939)

107. [Nieto-Castanon A, Whitfield-Gabrieli S. *CONN Functional Connectivity Toolbox: RRID SCR_009550, Release 22*. Hilbert Press; 2022.](http://paperpile.com/b/W6ksH6/FPKBW)

108. [Nieto-Castanon A. *Handbook of Functional Connectivity Magnetic Resonance Imaging Methods in CONN*. Hilbert Press; 2020.](http://paperpile.com/b/W6ksH6/kelyH)

109. [Henschel L, Conjeti S, Estrada S, Diers K, Fischl B, Reuter M. FastSurfer - A fast and accurate deep learning based neuroimaging pipeline. *Neuroimage*. 2020;219:117012.](http://paperpile.com/b/W6ksH6/PWlUg)

110. [Klein A, Tourville J. 101 labeled brain images and a consistent human cortical labeling protocol. *Front Neurosci*. 2012;6:171.](http://paperpile.com/b/W6ksH6/rKmIQ)

111. [Desikan RS, Ségonne F, Fischl B, et al. An automated labeling system for subdividing the human cerebral cortex on MRI scans into gyral based regions of interest. *Neuroimage*. 2006;31(3):968-980.](http://paperpile.com/b/W6ksH6/kAwDl)

112. [Tournier JD, Smith R, Raffelt D, et al. MRtrix3: A fast, flexible and open software framework for medical image processing and visualisation. *Neuroimage*. 2019;202:116137.](http://paperpile.com/b/W6ksH6/lTp23)

113. [Avants BB, Tustison NJ, Song G, Cook PA, Klein A, Gee JC. A reproducible evaluation of ANTs similarity metric performance in brain image registration. *Neuroimage*. 2011;54(3):2033-2044.](http://paperpile.com/b/W6ksH6/UhiW4)

114. [Jenkinson M, Beckmann CF, Behrens TEJ, Woolrich MW, Smith SM. FSL. *Neuroimage*. 2012;62(2):782-790.](http://paperpile.com/b/W6ksH6/GaTiG)

115. [Rubinov M, Sporns O. Complex network measures of brain connectivity: uses and interpretations. *Neuroimage*. 2010;52(3):1059-1069.](http://paperpile.com/b/W6ksH6/PFLXM)

116. [Wang Y, Ghumare E, Vandenberghe R, Dupont P. Comparison of Different Generalizations of Clustering Coefficient and Local Efficiency for Weighted Undirected Graphs. *Neural Comput*. 2017;29(2):313-331.](http://paperpile.com/b/W6ksH6/nzSTK)
